# Age-related vaginal microecology and infection epidemiology among premenopausal and postmenopausal gynecologic outpatients: a cross-sectional study

**DOI:** 10.3389/fcimb.2025.1569667

**Published:** 2025-08-04

**Authors:** Wenyu Lin, Liying Wang, Binhua Dong, Yuhang Zhang, Yan Zhang, Liang Wang, Jun Shen, Yanfang Lu, Meijin Zheng, Pengming Sun

**Affiliations:** ^1^ Laboratory of Gynecologic Oncology, Fujian Maternity and Child Health Hospital, College of Clinical Medicine for Obstetrics and Gynecology and Pediatrics, Fujian Medical University, Fuzhou, Fujian, China; ^2^ Fujian Key Laboratory of Women and Children’s Critical Diseases Research, Fujian Maternity and Child Health Hospital (Fujian Women and Children’s Hospital), Fuzhou, Fujian, China; ^3^ Fujian Clinical Research Center for Gynecological Oncology, Fujian Maternity and Child Health Hospital (Fujian Obstetrics and Gynecology Hospital), Fuzhou, Fujian, China; ^4^ The State Key Laboratory of Molecular Vaccinology and Molecular Diagnostics, National Institute of Diagnostics and Vaccine Development in Infectious Diseases, School of Public Health, Xiamen University, Xiamen, Fujian, China; ^5^ Fujian Provincial Cervical Disease Diagnosis and Treatment Health Center, Fujian Maternity and Child Health Hospital, Affiliated Hospital of Fujian Medical University, Fuzhou, Fujian, China; ^6^ Department of Clinical Laboratory, Fujian Maternity and Child Health Hospital, Affiliated Hospital of Fujian Medical University, Fuzhou, Fujian, China

**Keywords:** vaginal microecology, vaginal infection, menopause, microbiota, metabolites

## Abstract

**Background:**

Postmenopausal estrogen deficiency disrupts vaginal microecological balance. This cross-sectional study investigates the epidemiology of vaginal infections and alterations in microbiota composition, enzymes, and metabolites among premenopausal and postmenopausal gynecologic outpatients.

**Methods:**

The study analyzed the vaginal microecology data from 27,346 women who underwent examinations at Fujian Maternity and Child Health Hospital between 2018 and 2023. Parameters including vaginal cleanliness, bacterial density, and diversity were systematically evaluated. Additionally, a total of 20 participants (10 premenopausal and 10 postmenopausal women) were enrolled for nontargeted LC-MS metabolomic analysis through stratified random sampling.

**Results:**

The population comprised 22,525 (82.4%) premenopausal women (18–44 years), 3,456 (12.6%) transitioning women (45–55 years), and 1,365 (5.0%) postmenopausal women (>55 years). In mixed infections, BV + VVC co-infections predominated (1264/2766, 45.7%). Postmenopausal women showed significantly higher BV prevalence (22.8% *vs*. 17.9%, P < 0.001) and AV (24.8% *vs*. 4.6%, P < 0.001), but lower rates of VVC (1.2% *vs*. 8.2%, P < 0.001). In postmenopausal women, BV-associated biomarkers (including clue cells and sialidase activity) and inflammatory markers (such as pus cells and leukocyte esterase activity) were concurrently elevated. Metabolomic analysis identified elevated chenodeoxycholic acid glycine conjugate levels alongside reduced O-phosphothreonine, morpholine, and diethanolamine.

**Conclusion:**

Age significantly influences vaginal microecology, altering infection epidemiology, microbiota, enzymes, and metabolites. Accounting for these age-related estrogen changes in clinical interventions is critical for effective management.

## 1 Introduction

Vaginal microecology consists of the vaginal microbiota, immune environment, anatomy, and cervicovaginal fluid enriched with metabolites, enzymes, and cytokines (Brotman et al., 2018; Ye and Qi, 2024). The vaginal microbiota plays a pivotal role in preventing pathogen colonization and maintaining gynecologic and reproductive health (Witkin and Linhares, 2017). Vaginal microecology is dynamic and influenced by various endogenous and exogenous factors, including age, diet, behavior, health, ethnicity, sexual habits, and gynecological or urinary health (Kaur et al., 2020). Fluctuations in estrogen levels during different reproductive stages (puberty, menstruation, pregnancy, and menopause) significantly alter the vaginal microecology (Kaur et al., 2020). Despite the high prevalence of vaginal microbiota disorders in postmenopausal women, this health concern remains inadequately addressed.

In postmenopausal women, diminished estrogen levels induce vaginal epithelial atrophy accompanied by a pronounced reduction or complete depletion of glycogen reserves. This hormonal decline directly compromises *Lactobacillus* dominance, elevates vaginal pH, and promotes enterobacterial colonization (Petricevic et al., 2013). A comparative cohort study of 70 pre- and postmenopausal women (Yoshikata et al., 2022) revealed significantly reduced *Lactobacillus* abundance, heightened microbial diversity, and increased vaginal pH in postmenopausal women. However, these findings should be interpreted cautiously due to the small sample size and lack of assessments for postmenopausal vaginal infections.

Vaginal infections encompass bacterial vaginosis (BV), aerobic vaginitis (AV), vulvovaginal candidiasis (VVC), trichomonas vaginitis (TV), and cytolytic vaginosis (CV). These conditions collectively account for up to 50% of gynecologic consultations and impose substantial economic burdens on healthcare systems (Mitra et al., 2016). BV is characterized by vaginal microbiota dysbiosis, featuring elevated levels of *Gardnerella vaginalis (GV)*, *Atopobium, Prevotella, Sneathia, Peptostreptococcus, Megasphaera*, and BV-associated bacteria (BVAB1–BVAB3), alongside reduced *Lactobacilli* spp (Muzny et al., 2019). Diagnosis relies on the Nugent score (microscopic bacterial morphotypes) or the Amsel criteria (requiring ≥3 of the following: thin discharge, elevated pH, amine odor, or clue cells in wet mount microscopy) (Cauci et al., 2002). CV results from *Lactobacilli* overgrowth, inducing vaginal squamous epithelial cell lysis and symptomatic manifestations (Xu et al., 2019). VVC, caused by *Candida* sp*ecies*, is characterized by white, viscous discharge resembling tofu residue and intense vulvar itching (Gonçalves et al., 2016). TV, caused by *Trichomonas vaginalis*, is confirmed through nucleic acid amplification tests (NAATs). This pathogen colonizes both genital and urinary tracts, provoking urethritis or cystitis (Schwebke et al., 2024). AV arises from diminished Lactobacilli and proliferation of aerobic bacteria. The heightened microbial diversity and pathogenic complexity associated with AV frequently lead to mixed infections (Ma et al., 2022).

Hormonal or environmental shifts in cervicovaginal microbial communities alter the metabolome. For instance, non-*Lactobacillus* dominant communities, particularly in high-grade dysplasia, perturb amino acid and nucleotide metabolisms (Nelson et al., 2015; Ilhan et al., 2019). Investigating microbe-metabolite crosstalk in the reproductive tract is crucial for understanding inflammation, adverse pregnancy outcomes, tumorigenesis, and for identifying diagnostic and therapeutic targets (Li et al., 2020). Nevertheless, the relevance of cervicovaginal metabolites, particularly those associated with the age or menopause, remains insufficiently elucidated.

Existing epidemiological evidence demonstrates that hormonal status—a key determinant of women’s health trajectories throughout life stages—profoundly influences vaginal ecosystem dynamics (Brotman et al., 2018). Therefore, we constructed a cohort of 27,346 individuals in the for clinical vaginal microecology analysis, supplemented by untargeted metabolomic analysis of 20 vaginal discharge specimens. We aimed to investigate the epidemiology of age-stratified vaginal infections in pre- and postmenopausal women through a hospital-based cross-sectional study, and characterize microbiota composition, enzymes, and metabolites alterations.

## 2 Materials and methods

### 2.1 Study population

**Figure 1:**
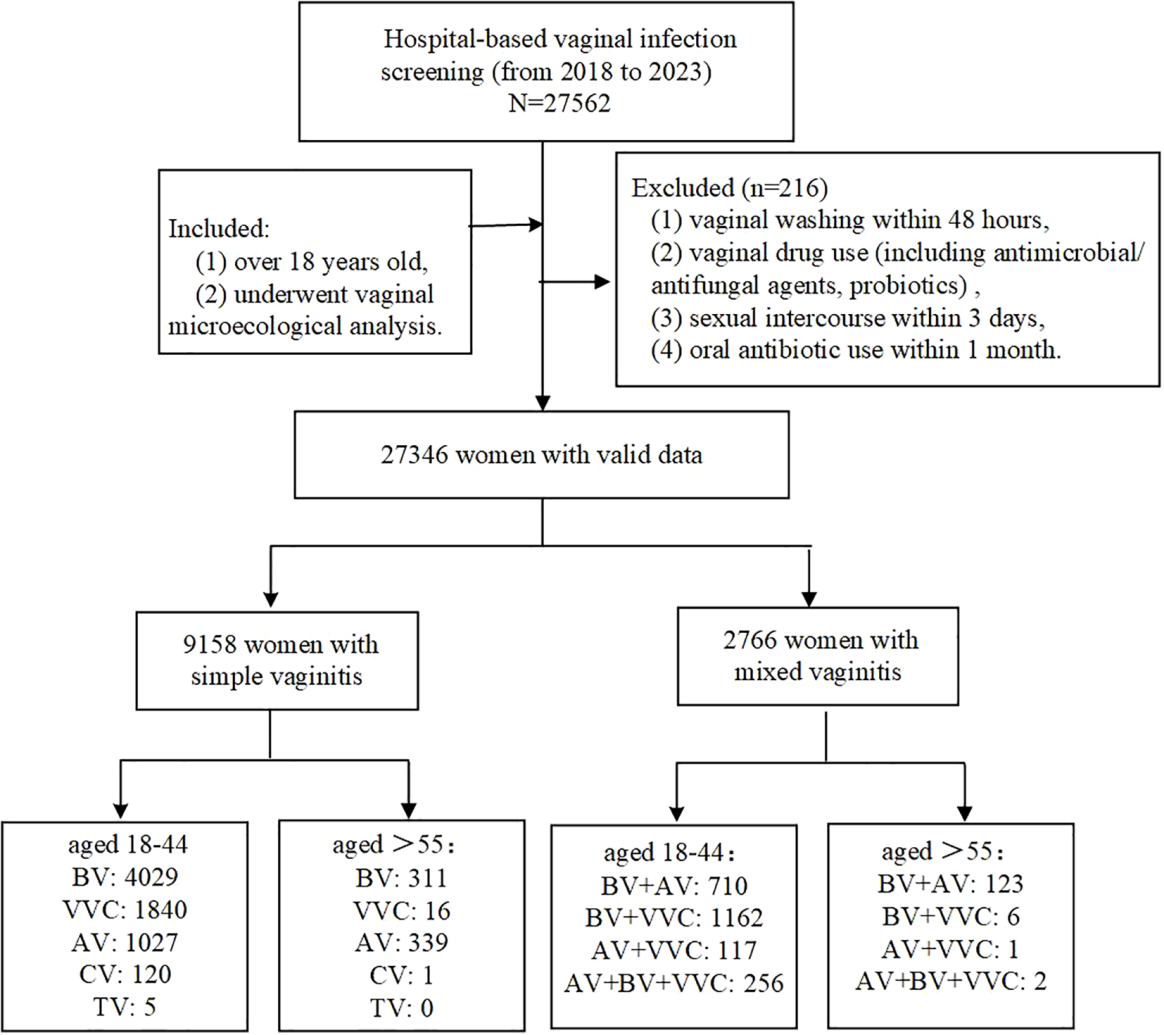
Flow chart.

This study enrolled 27,346 participants from Fujian Maternity and Child Health Hospital, affiliated with Fujian Medical University, between April 2018 and July 2023 (**Figure 1**). Participants met the inclusion criteria if they were (1) over 18 years old, (2) underwent vaginal microecological analysis, with the cohort comprising symptomatic women (e.g., vaginitis), asymptomatic individuals undergoing routine gynecological screening, and pre-procedural evaluation for assisted reproductive technology (ART) or gynecological surgeries. Exclusion criteria included: (1) vaginal washing within 48 hours, (2) vaginal drug use (including antimicrobial/antifungal agents, probiotics), (3) sexual intercourse within 3 days, and (4) oral antibiotic use within 1 month. Participants were stratified into premenopausal (18–44 years), menopausal transition (45–55 years), and postmenopausal (>55 years) groups based on age-defined reproductive status (Wang et al., 2021; Menopause Study Group et al., 2023). Based on the above-described premenopausal and postmenopausal populations, a total of 20 participants (10 premenopausal and 10 postmenopausal women) were enrolled via stratified random sampling for non-targeted LC-MS metabolomic analysis. This study was approved by the Ethics Committee of Fujian Maternity and Child Health Hospital (2020KY148). All experiments followed relevant regulations and were conducted under Ethics Committee supervision.

### 2.2 Sample collection

Sterile cotton swabs collected vaginal discharge from the upper third of the vaginal wall with 10–15 seconds of rotation. Samples were treated using the pre-treatment solution of the Unit-700 vaginal microecological analyzer (Unit-700, Shtars, China). The swab was immersed in the pre-treatment solution, and the tube walls were gently squeezed to ensure thorough dissolution of secretions into the liquid. Automated detection was then performed using the Unit-700 vaginal microecological analyzer coupled with the Comet 800 microscopic examination and analysis system.

### 2.3 Vaginal microecology analysis

#### 2.3.1 Microscopic examination of brine wet film

1. Slice preparation: The collected vaginal secretion sample was applied directly and evenly onto a slide that was dripping with normal saline.
2. Microscopic examination: the presence or absence of *Trichomonas* and *Candida* hyphae were observed under low magnification, while the number of leukocytes, epithelial cells, bacilli and cocci was observed under high magnification to identify the presence of *Candida* bacteria and spores.

#### 2.3.2 Evaluation criteria

Vaginal PH value was determined by color strips. Vaginal pH: normal, pH ≤ 4.5; abnormal, pH > 4.5. Vaginal cleanliness was classified according to the National Clinical Laboratory Practice Guidelines (18th edition): grades I–II were considered normal, while grades III–IV were classified as abnormal. The bacterial diversity was graded as follows (Cooperative Group of Infectious Disease Chinese Chinese Society of Obstetrics and Gynecology Chinese Medical Association, 2016): level I (+) was defined as 1–3 bacterial species identified per oil immersion microscope field of view; level II (++), 4–6 species; level III (+++), 7–9 species; and level IV (++++), ≥10 species. Normal microbiota status was defined as diversity and density scores between ++ and +++, with deviations from this range indicating dysbiosis. Sialidase colorless is normal (-), red or purple is positive (+). LE colorless is normal (-), and green or blue is positive (+). H_2_O_2_>2 mmol/L is red or purple, negative (-), H_2_O_2_<2 mmol/L is positive (+), blue.

Diagnostic evaluations were performed based on the Vaginal Microecology Evaluation System (version 2016) (Cooperative Group of Infectious Disease Chinese Chinese Society of Obstetrics and Gynecology Chinese Medical Association, 2016), as follows: (1) Normal vaginal microecology: density and diversity grades were II–III, the dominant bacteria were *Lactobacillus*, no pathogenic microorganisms were detected, vaginal pH value was 3.8–4.5, H_2_O_2_ was positive, leukocyte esterase and sialidase were negative; (2) BV: Nugent score ≥7 points; (3) VVC: fungal spores or pseudohyphae could be found microscopically under oil; (4) AV: according to the clinical manifestations and microscopic Donders score ≥3 points; (5) Trichomoniasis: a large number of white blood cells and active Trichomoniasis under the microscope; (6) Mixed vaginitis: two or more types of vaginitis existing at the same time. All laboratory procedures were conducted according to the manufacturer’s instructions.

### 2.4 Metabolite extraction

The samples were thawed slowly at 4°C, and metabolites were extracted using 300 μL of methanol: acetonitrile (2:1, v:v) at –20°C for 2 h. The samples were then centrifuged at 4000 g for 20 min at 4°C. After centrifugation, 300 μL of supernatant was dried with a vacuum concentration meter and then redissolved in 150 μL methanol:H2O (1:1, v:v). Then, the samples were centrifuged at 4000 g for 30 min at 4°C. Ten microliters of supernatant was mixed as a quality control (QC) sample.

### 2.5 Data processing and metabolite identification

Vaginal secretion samples were eluted from swabs using methanol and subjected to nontargeted liquid chromatography-mass spectrometry (LC-MS)-based metabolomics conducted by BI TREE (Shanghai, China). Briefly, LC-MS was performed using ultra-performance liquid chromatography (Waters 2D UPLC; Waters, Milford, USA) and high-resolution mass spectrometry (Thermo Fisher Scientific, Waltham, USA), and sSamples were analyzed in positive and negative ion modes. Features in the LC–MS metabolomics raw data were aligned, and peak areas were determined using XCMSonline (https://xcmsonline.scripps.edu/). Statistical analysis was performed by an online tool called MetaboAnalyst 4.0 (http://www.metaboanalyst.ca). Hierarchical clustering was used to detect the classification ability and concentration levels of metabolites. Log transmission and autoscaling were used on metabolomics data to make the data obey a normal distribution for drawing a heatmap. The Kruskal–Wallis test of the original data was used for pairwise comparisons. Chemometrics analysis of principal component analysis (PCA) was applied to reveal the global metabolic difference of different groups and evaluate the stability and credibility.

### 2.6 Statistical analysis

The data were calculated using the IBM SPSS statistical package version 22.0 (IBM, Corporation, Armonk, USA). Categorical variables were expressed as numbers (N) and percentages (%), and continuous variables were expressed as medians. Comparisons between premenopausal and postmenopausal women were performed using the chi-squared test and Fisher’s exact test for proportions. The significance level was set at a two-tailed p-value < 0.05.

## 3 Results

### 3.1 Characteristics of the study population

Demographic and clinical characteristics of the cohort (N=27,346) are summarized in **Table 1**. The population comprised 22,525 (82.4%) premenopausal women (18–44 years), 3,456 (12.6%) transitioning women (45–55 years), and 1,365 (5.0%) postmenopausal women (>55 years). Vaginal microbiological profiles indicated that 15,422 women (56.4%) had normal vaginal microbiology, while 9,158 (33.5%) presented with simple infections and 2,766 (10.1%) had mixed infections.

**Table 1:** Baseline characteristics of adult participants (N=27346).

Characteristics	Case, n	Proportion, %	
Age			
18-44	22525	82.4%	
45-55	3456	12.6%	
>55	1365	5.0%	
Vaginal microecology test			
Normal	15422	56.4%
Abnormal	11924	43.6%
Simple vaginitis			
BV	5336	44.8%
VVC	2013	16.9%
AV	1674	14.0%
TV	6	0.0%
CV	129	1.1%
Mixed vaginitis			
BV+AV	1100	9.2%
BV+VVC	1264	10.6%
AV+VVC	128	1.1%
AV+BV+VVC	274	2.3%
Cleanliness levels			
Normal	11359	41.5%	
Abnormal	15987	58.5%	
PH			
Normal	10702	39.1%	
Abnormal	16644	60.9%	
Bacterial density			
Normal	21584	78.9%	
Abnormal	5762	21.1%	
Bacterial diversity			
Normal	25316	92.6%	
Abnormal	2030	7.4%	
Leukocyte			
Negative	103	0.4%	
Positive	27243	99.6%	
Pus Cells			
Negative	7120	26.0%	
Positive	20226	74.0%	
Erythrocytes			
Negative	23268	85.1%	
Positive	4078	14.9%	
Clue cells			
Negative	21718	79.4%	
Positive	5628	20.6%	
Catalase			
Negative	4386	16.0%	
Positive	22960	84.0%	
Sialidase			
Negative	22505	82.3%	
Positive	4841	17.7%	
Leukocyte esterase			
Negative	6059	22.2%	
Positive	21287	77.8%	
Dominant bacteria			
Gram-Positive Rods	15687	57.4%	
Gram-Positive Cocci	1320	4.8%	
Gram-Negative Rods	8113	29.7%	
Gram-Negative Cocci	5	0.0%	
Gram-Negative Vibrio	6	0.0%	
*Gardnerella/Prevotella*	436	1.6%	
*Lactobacillus*	1	0.0%	
Others	632	2.3%	
Not Found	1146	4.2%	

Among single infections (**Table 2**), BV (5336/9158, 58.3%) was most prevalent, followed by VVC (2013/9158, 22.0%), AV (1674/9158, 18.3%), CV (129/9158, 1.4%), and TV (6/9158, 0.0%). In mixed infections, BV + VVC co-infections predominated (1264/2766, 45.7%). Age-stratified analysis demonstrated BV prevalence peaked in the 18 - 44 (43.4%) and 45 - 55 (53.6%) cohorts, whereas AV incidence was highest in the >55-year-old group (42.4%).

**Table 2:** The infection status of simple vaginitis and mixed vaginitis.

Infection status, n(%)	18-44 (n=9266)	45-55 (n=1859)	>55 (n=799)
Simple vaginitis (n=9158)			
BV (n=5336)	4029 (43.4%)	996 (53.6%)	311 (38.9%)
VVC (n=2013)	1840 (19.9%)	157 (8.4%)	16 (2.0%)
AV (n=1674)	1027 (11.1%)	308 (16.6%)	339 (42.4%)
TV (n=6)	5 (0.0%)	1 (0.0%)	0 (0.0%)
CV (n=129)	120 (1.3%)	8 (0.4%)	1 (0.1%)
Mixed vaginitis (n=2766)			
BV+AV (n=1100)	710 (7.7%)	267 (14.4%)	123 (15.4%)
BV+VVC (n=1264)	1162 (12.5%)	96 (5.2%)	6 (0.8%)
AV+VVC (n=128)	117 (1.3%)	10 (0.5%)	1 (0.1%)
AV+BV+VVC (n=274)	256 (2.8%)	16 (0.9%)	2 (0.3%)

Mixed vaginitis: two or more types of vaginitis existing at the same time. BV, Bacterial Vaginosis; VVC, Vulvovaginal Candidiasis; AV, Aerobic Vaginitis; TV, Trichomonas Vaginalis; CV, Cytolytic Vaginosis.

### 3.2 Vaginal infections in pre- and postmenopausal women

To assess age-related differences in vaginitis infections, chi-square tests were performed for five infection types across groups (**Table 3**). Postmenopausal women showed significantly higher BV prevalence (22.8% *vs*. 17.9%, P < 0.001) and AV (24.8% *vs*. 4.6%, P < 0.001), but lower rates of VVC (1.2% *vs*. 8.2%, P < 0.001). Although CV exhibited intergroup differences (P = 0.005), interpretation requires caution due to limited sample sizes. No significant association was observed for TV (P=0.828).

**Table 3:** Infection status of simple vaginitis in pre- and post-menopausal populations.

Infection Status, n(%)	18-44 (n=22525)	45-55 (n=3456)	>55 (n=1365)	*χ^2^*	*P*
BV				237.814	<0.001
Negative (n=22010)	18496 (82.1%)	2460 (71.2%)	1054 (77.2%)		
Positive (n=5336)	4029 (17.9%)	996 (28.8%)	311 (22.8%)		
VVC				138.466	<0.001
Negative (n=25333)	20685 (91.8%)	3299 (95.5%)	1349 (98.8%)		
Positive (n=2013)	1840 (8.2%)	157 (4.5%)	16 (1.2%)		
TV				0.377	0.828
Negative (n=27340)	22520(100.0%)	3455(100.0%)	1365 (100.0%)		
Positive (n=6)	5 (0.0%)	1 (0.0%)	0 (0.0%)		
AV				974.283	<0.001
Negative (n=25672)	21498 (95.4%)	3148 (91.1%)	1026 (75.2%)		
Positive (n=1674)	1027 (4.6%)	308 (8.9%)	339 (24.8%)		
CV				10.651	0.005
Negative (n=27217)	22405 (99.5%)	3448 (99.8%)	1364 (100.0%)		
Positive (n=129)	120 (0.5%)	8 (0.2%)	1 (0.0%)		

BV, Bacterial Vaginosis; VVC, Vulvovaginal Candidiasis; AV, Aerobic Vaginitis; TV, Trichomonas Vaginalis; CV, Cytolytic Vaginosis.

### 3.3 Vaginal microenvironment in pre- and postmenopausal women

Further comparative analysis of vaginal microenvironment parameters revealed that menopausal status (age > 55 years) significantly impacted these parameters (**Table 4**). Postmenopausal women demonstrated significantly elevated abnormality rates in bacterial density (χ² = 879.895, P<0.001) and diversity (χ² = 1695.280, P<0.001). BV-associated biomarkers, including clue cells (χ² = 246.178, P < 0.001) and sialidase activity (χ² = 246.407, P< 0.001), were markedly increased. Concurrently, inflammatory markers such as pus cells (χ² = 43.299, P < 0.001) and leukocyte esterase activity (χ² = 284.800, P < 0.001) showed marked elevation.

**Table 4:** Comparison of vaginal microenvironment changes in pre- and post-menopausal women.

Characteristics, n(%)	18-44 (n=22525)	45-55 (n=3456)	>55 (n=1365)	*χ^2^*	*P*
Cleanliness Levels				115.302	<0.001
Normal (n=11359)	9682 (43.0%)	1238 (35.8%)	439 (32.2%)		
Abnormal (n=15987)	12843 (57.0%)	2218 (64.2%)	926 (67.8%)		
PH				789.763	<0.001
Normal (n=10702)	9627 (42.7%)	918 (26.6%)	157 (11.5%)		
Abnormal (n=16644)	12898 (57.3%)	2538 (73.4%)	1208 (88.5%)		
Bacterial Density				879.895	<0.001
Normal (n=21584)	18484 (82.1%)	2366 (68.5%)	734 (53.8%)		
Abnormal (n=5762)	4041 (17.9%)	1090 (31.5%)	631 (46.2%)		
Bacterial Diversity				1695.280	<0.001
Normal (n=25316)	21356 (94.8%)	3066 (88.7%)	894 (65.5%)		
Abnormal (n=2030)	1169 (5.2%)	390 (11.3%)	471 (34.5%)		
Leukocyte				20.016	<0.001
Negative (n=103)	77 (0.3%)	11 (0.3%)	15 (1.1%)		
Positive (n=27243)	22448 (99.7%)	3445 (99.7%)	1350 (98.9%)		
Pus Cells				43.299	<0.001
Negative (n=7120)	6344 (28.1%)	530 (15.3%)	246 (18.0%)		
Positive (n=20226)	16181 (71.9%)	2926 (84.7%)	1119 (82.0%)		
Erythrocytes				2.141	0.343
Negative (n=23268)	19774 (87.8%)	2534 (73.3%)	960 (78.1%)		
Positive (n=4078)	2751 (12.2%)	922 (26.7%)	405 (21.9%)		
Clue Cells				246.178	<0.001
Negative (n=21718)	18251 (81.0%)	2401 (69.4%)	1066 (78.1%)		
Positive (n=5628)	4274 (19.0%)	1055 (30.6%)	299 (21.9%)		
Catalase				188.590	<0.001
Negative (n=4386)	3918 (17.4%)	379 (11.0%)	89 (6.5%)		
Positive (n=22960)	18607 (82.6%)	3077 (89.0%)	1276 (93.5%)		
Sialidase				246.407	<0.001
Negative (n=22505)	18899 (83.9%)	2531 (73.2%)	1075 (78.8%)		
Positive (n=4841)	3626 (16.1%)	925 (26.8%)	290 (21.2%)		
Leukocyte Esterase				284.800	<0.001
Negative (n=6059)	5404 (24.0%)	547 (15.8%)	108 (7.9%)		
Positive (n=21287)	17121 (76.0%)	2909 (84.2%)	1257 (92.1%)		

### 3.4 Metabolite differences in pre- and postmenopausal women

**Figure 2:**
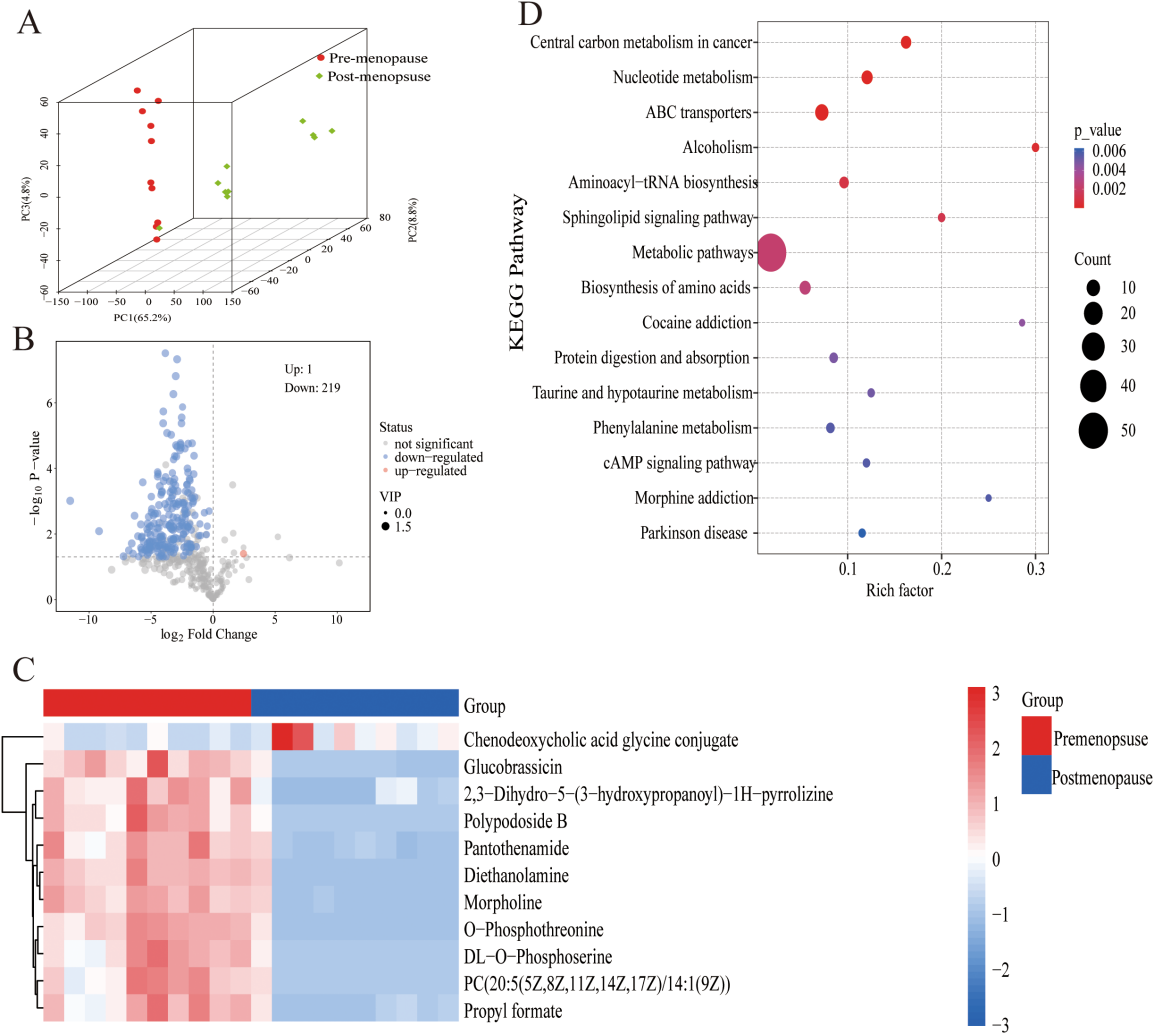
Metabolite differences in pre- and postmenopausal women. **(A)** Significantly regulated metabolites between pre-menopause and post-menopause groups. **(B)** The DEGs between pre-menopause and post-menopause groups. **(C)** Significantly regulated metabolites between pre-menopause and post-menopause groups were shown with heat maps. **(D)** Functional enrichment analysis of DEGs.

A comprehensive metabolomic landscape was obtained using LC-MS-based analysis of vaginal discharge samples. The PCA model visualization results (**Figure 2A**) revealed distinct compositional differences in vaginal metabolites between pre- and postmenopausal women. Screening thresholds for differential metabolites were defined as fold change (FC) > 2 or < 0.5 with P < 0.05. Metabolite profiling identified 219 downregulated metabolites and 1 upregulated metabolite in the postmenopausal vaginal microenvironment (**Figure 2B**). **Figure 2C** shows the first 10 differential metabolites. Levels of nine metabolites, such as O-Phosphothreonine, Morpholine, and Diethanolamine, were decreased in postmenopausal women. Conversely, chenodeoxycholic acid glycine conjugate levels were moderately increased in postmenopausal women. The KEGG analysis showed that significantly differential pathways included Central carbon metabolism in cancer, Nucleotide metabolism, ABC transporters, Alcoholism, etc (**Figure 2D**). These findings collectively illustrate dynamic metabolic shifts associated with the menopause.

## 4 Discussion

Age-related decline in estrogen affects the vaginal microecology. Our findings reveal a postmenopausal shift characterized by elevated incidences of BV and AV, contrasted with reduced prevalence of VVC. Metabolomic profiling further indicates a generalized downregulation of vaginal metabolic activity postmenopause, with henodeoxycholic acid glycine conjugate emerging as a prominently upregulated metabolite.

Our research revealed an increased incidence of BV among postmenopausal women, a phenomenon warranting further investigation. BV is characterized by the depletion or significant reduction of *Lactobacillus*, accompanied by a marked proliferation of anaerobic microbes (Zhu et al., 2024). *Gardnerella vaginalis*, an anaerobic pathogen, has been identified as the primary etiological agent of BV. *Gardnerella* possesses several virulence factors that can contribute to its pathogenic phenotype, including a hemolysin, mucus-degrading sialidases, resistance factors, and biofilm formation (Qin and Xiao, 2022). *Gardnerella*-derived biofilms have been strongly implicated in BV recurrence (Li et al., 2024). *Gardnerella* sialidases cleave terminal sialic acid residues off of human glycans (Chen et al., 2024). By desialylation, sialidases not only alter the function of sialic acid-containing glycoconjugates but also play a vital role in the attachment, colonization and spread of many other vaginal pathogens.

Liu Zhaohui’s study employed metagenomic detection to investigate the vaginal microbiota composition in postmenopausal and postpartum women. The study revealed that the dominance of *Lactobacillus* decreased, whereas microbial diversity increased significantly in postmenopausal and postpartum women (Li et al., 2024). In the vaginal microbiota of premenopausal women, *Lactobacillus* species constituted approximately 71.98%, and pathogenic flora constituted approximately 16.87%. In contrast, postmenopausal women exhibited reduced proportions of *Lactobacillus* (10.08%) and elevated pathogenic flora (26.78%) (Yoshikata et al., 2022). Postmenopausal hormonal depletion diminishes vaginal epithelial glycogen levels, thereby limiting metabolic substrates essential for *Lactobacillus* proliferation. Furthermore, *Lactobacillus* suppresses pathogen colonization through competitive exclusion, inhibition of epithelial adhesion, and secretion of antimicrobial compounds (e.g., bacteriocins, lactic acid) and immunomodulatory agents. However, further metagenomics techniques are needed to identify changes in the proportion of different *Lactobacillus* species in the postmenopausal vaginal microenvironment.

VVC susceptibility exhibits an age-dependent decline, consistent with our observations. This phenomenon is mechanistically linked to elevated vaginal pH and diminished glycogen levels in postmenopausal women, which collectively restrict *Candida* proliferation (Zhao et al., 2022).

For menopausal-associated BV and VVC, therapeutic interventions frequently require combination therapy (Dothard et al., 2023; Li et al., 2024). Estrogen therapy has demonstrated clinical efficacy in addressing menopause-associated VVC and BV by restoring vaginal epithelial integrity and glycogen reserves (Sookkhee et al., 2024). Gliniewicz et al. conducted a comparative analysis of vaginal microbiota profiles, contrasting premenopausal women with postmenopausal individuals receiving hormone replacement therapy (HRT). By measuring 16s rRNA gene copies, the researchers determined that postmenopausal women receiving HRT were most often dominated by species of *Lactobacillus*, and had similar bacterial counts to premenopausal women (Gliniewicz et al., 2019). Furthermore, emerging as a complementary therapeutic modality, probiotic supplementation shows considerable potential for correcting vaginal dysbiosis. Clinical trials report that probiotic-treated patients exhibit microbiota shifts favoring *Lactobacillus* colonization (Burton et al., 2003).

The metabolites produced within the female reproductive tract play a pivotal role in the pathogenesis of female genital tract inflammation, and may serve as biological markers for disease severity, diagnosis, and prognosis. Metabolites are primarily amino acids, carbohydrates, and lipids, and are closely associated with physiological processes. We conducted a metabolomic sequencing of vaginal secretions from ten premenopausal and ten postmenopausal women, and observed differential expression levels of ten metabolites. The carbohydrates include polypodoside B, while the amino acids encompass glucobrassicin, pantothenamide and O-phosphothreonine. The lipids comprise chenodeoxycholic acid. Additionally, the presence of acid glycine conjugate and PC(20:5(5Z,8Z,11Z,14Z,17Z)/14:1(9Z)) propyl formate was identified. These metabolites exhibited differential expression in the aforementioned metabolic differences. In the aforementioned metabolic profile, only the lipid chenodeoxycholic acid glycine conjugate demonstrated an increase in concentration in the metabolic profile of the premenopausal female subjects.

The vaginal microenvironment shares many similarities with the intestinal microenvironment in that the flora interacts with the vaginal/intestinal microenvironment, the vagina/intestinal tract provides the microorganisms with the nutrients needed for their own reproduction, while at the same time the microorganisms produce a large number of metabolites as a result of their interactions with the host. There has been a great deal of research on Chenodeoxycholic acid in the gut microecology (Cai et al., 2022), Chenodeoxycholic acid (CDCA), a primary bile acid, significantly regulates local immunity via several mechanisms. First, CDCA activates the farnesoid X receptor (FXR), which suppresses pro-inflammatory cytokines such as TNF-α, IL-6, and IL-1β, thereby attenuating inflammation. Second, CDCA facilitates the development of RORγ+ regulatory T cells (Tregs), which are crucial for maintaining immune balance and suppressing inflammation. Third, CDCA modulates glucagon-like peptide-1 (GLP-1) expression and secretion. GLP-1 not only governs glucose metabolism but also has immunomodulatory functions (Rau et al., 2016; Fiorucci et al., 2018).

The advantage of this study lies in the large sample size and the utilization of metabolomics research methods. Several study limitations should be noted. First, our hospital-based study may not fully represent community populations, future multicenter studies incorporating community-based sampling strategies are warranted to validate the generalizability of these findings. However, this hospital-based study design ensured standardization of diagnostic procedures. Secondly, the retrospective data resulted in incomplete documentation of key covariates, including Symptoms and signs of vaginitis, oral contraceptive use, HRT, and hygiene practices (Daher et al., 2022). Future studies should incorporate prospective longitudinal tracking of these variables—including detailed contraceptive/HRT exposure histories, standardized symptom assessments, and hygiene behavior monitoring—with stratified analyses to elucidate their mechanistic roles.

Vaginitis in premenopausal and postmenopausal women differs significantly, as do their metabolic mechanisms. Consequently, clinical management of postmenopausal vaginal infections demands specialized approaches. Beyond pathogen-directed antimicrobial therapy, restoration of vaginal microecological balance through adjunctive interventions such as topical estrogen or probiotic supplementation should be prioritized to address the hypoestrogenic microenvironment characteristic of postmenopausal women.

## Data Availability

The original contributions presented in the study are included in the article/supplementary material. Further inquiries can be directed to the corresponding authors.
